# Chiropractic diagnosis and management of non-musculoskeletal conditions in children and adolescents

**DOI:** 10.1186/1746-1340-18-14

**Published:** 2010-06-02

**Authors:** Randy J Ferrance, Joyce Miller

**Affiliations:** 1 Hospitalist and Medical Director of Hospital-Based Quality, Riverside Tappahannock Hospital, Tappahannock, VA, USA; 2 Associate Professor, Anglo-European College of Chiropractic, Bournemouth, UK

## Abstract

**Background:**

A great deal has been published in the chiropractic literature regarding the response, or lack thereof, of various common pediatric conditions to chiropractic care. The majority of that literature is of low scientific value (that is, case reports or case series). The purpose of this review is to summarize the literature from the point of view of clinicians, rather than researchers, and to discuss some additional detail of the conditions themselves.

**Methods:**

Databases searched were PubMed, Mantis, Index to Chiropractic Literature, and CINAHL. Keywords were chiropractic paired with colic, crying infant, nocturnal enuresis, asthma, otitis media and attention deficit hyperactivity disorder.

**Results:**

Most of the published literature centers around case reports or series. The more scientifically rigorous studies show conflicting results for colic and the crying infant, and there is little data to suggest improvement of otitis media, asthma, nocturnal enuresis or attention deficit hyperactivity disorder.

**Discussion:**

The efficacy of chiropractic care in the treatment of non-musculoskeletal disorders has yet to be definitely proven or disproven, with the burden of proof still resting upon the chiropractic profession.

## Background

While most patients presenting to a chiropractor's practice for care do so for musculoskeletal complaints, the National Center for Health Statistics found that in the United States, attention deficit hyperactivity disorder, sleep problems, asthma and sinusitis were also frequent complaints for which parents sought complementary and alternative medicine (CAM) for their children [1]. Children who are taken to a CAM practitioner tend to have underlying chronic medical problems and take medication on an ongoing basis, and chiropractors are the most common CAM providers visited by children and adolescents [2]. A recent systematic review found that chiropractors treat a wide variety of pediatric health conditions, but that those interventions are supported by only low levels of scientific evidence, most of which is clinical experience, descriptive case studies and very few observational and experimental studies [3]. While by no means comprehensive, this paper aims to list the conditions for which a child or adolescent patient might present for care in the hope of summarizing the currently available diagnostic criteria and evidence for chiropractic treatment.

## Review: Common Pediatric Conditions

### The Crying Infant

The excessively crying infant has been an enigmatic condition since it was first described by Spock in 1944 [4]. It continues to be the most common cause for medical consultation for infants under 16 weeks of age [5,6]. Multiple types of therapies have been proposed but few have withstood the rigors of scientific study. Conventional medicine has provided no answer to the problem of infant colic [7]. No medical treatments have been found to be effective except the medication, dicyclomine, which reduced crying in 63% of infants but was accompanied by side effects of apnea, seizures and coma and this treatment is now considered to be contraindicated in infants under six months of age [8].

Chiropractors have long claimed to provide an effective treatment for infant colic. In fact, a Best Evidence Report published in 2002 in the prestigious journal, Archives of Disease in Childhood, makes the statement, "There is good evidence that taking a colicky infant to a chiropractor will result in fewer reported hours of colic by parents [9]." However, it is not known whether the reduced crying is due to the specific therapy or because of the social setting surrounding the situation of taking a colicky baby to a unique practitioner [10]. There are two studies ongoing at this time in the UK and Denmark that will attempt to answer this question [7]. Meanwhile, we are left with the studies that have been done to test the efficacy of care for infant colic. Table 1 shows the studies that have been done in manual therapy with individual study results [11-15]. Most trials were poorly designed and poorly executed. If we are to find an effective treatment for infant colic, research must be improved to a threshold to be able to identify a worthy treatment, if it exists. Of importance in reviewing the risk/benefit ratios of available therapies, there were no adverse side effects in any of the manual therapy trials. Manual therapy may not have been proven to be effective, but it does appear to be safe [11-17].

**Table 1:** Summary of infant colic and manual therapy RCTs

Authors	N	Age and treatment numbers, type of treatment	Instrument	Therapy/Control	SSD	Level of evidence
Mercer and Nook	30	0 to 8 weeks of age; maximum of 6 treatments in 2 weeks, manual therapy (MT)	Subjective parent report questionnaires before treatment and subsequent visits	Chiropractic MT to spinal fixations v control of non functional detuned ultrasound machine	yes	C

Wiberg, Nordsteen and Nilsson	50	Treatment mean age 4.9; control mean age 5.9; 3-5 treatments over 2 weeks; manual therapy treatment	Validated crying diary	Light pressure with the finger tips v semithicone as control	yes	C

Olafsdottir, Forshei, Fluge and Markestad	100	3 to 9 wks of age, maximum of 3 treatments over maximum of 8 days; Manual therapy treatment	Crying diary and questionnaire at each visit. At the end of the observation period, the parents were contacted by telephone and interviewed	Light fingertip pressure on thoracic spine. Control infants were held by the nurse for 10 minutes with light back massage after being partially undressed in a similar way as treated infants	No; both groups had a mean reduction in crying from 5.1-5.4 to 3.1 hours per day	C

Koonin, Karpelow-sky, Yelverton and Brent-Rubens	31	Treatment mean age 5.7 weeks;control mean age 5.9 wks; 6 treatments maximum over 2 weeks; manual therapy treatment	Pre, post and follow up questionnaire	Chiropractic manual therapy with allopathic medication v allopathic medication alone	yes	C

Hayden and Mullinger	28	Treatment mean 6.6 weeks of age, control mean age 6.4 weeks; 2-4 treatments of manual therapy	Diary	Cranial osteopathic manual therapy v no treatment	yes	C

SSD-statistically significant difference; N-number of patients; C-level of evidence defined by Bronfort 1997

The trials that demonstrate effectiveness for manual therapy have significant weaknesses. Mercer and Nook, who presented their work at the 1999 World Federation of Chiropractic Conference failed to show clear methods of randomization and did not use the gold standard outcomes measure of the crying diary. It should be noted that this study has never been published in a peer review journal.

Wiberg et al (1999) found that chiropractic manipulation was effective in relieving infant colic. They used crying diaries and randomization; however parents were not blinded to the therapy received by the baby. This study was classified as "high quality" by Hawk et al using Jadad scoring as was the Olafsdottir et al (2001) study [18]. Olfasdottir et al in their randomized controlled trial found that chiropractic spinal manipulation was no better than placebo in the treatment of infant colic. In this study the investigators did not use a crying diary as an outcome measure, but instead asked the parent whether or not the child had improved and how much. This study has been criticized because it administered a maximum of three treatments whereas other trials used a more pragmatic approach prescribing the number of treatments the chiropractor found to be indicated within a 14 day time period. However, when comparing the Wiberg and Olafsdottir studies there was very little difference in the mean number of visits given (3 versus 3.8). The Olafsdottir trial was shorter with a maximum of eight days intervention. Nevertheless, the Olafsdottir study showed no significant difference in recovery between the group of infants that received pediatric manipulative therapy versus those who were held by the nurse with back massage. Koonin et al. did not use randomization and all children were on medication so it was a study of chiropractic care with medication versus treatment with medication only. This was a pragmatic study and may demonstrate that realistically, many infants who come for chiropractic care for infant colic are already using a medication; 45% of those in a crying study were taking medication when presenting to a chiropractic teaching clinic [19]. Hayden and Mullinger in an osteopathic study used an uncommon definition of colic crying of only 1.5 hours per day (colic is usually defined as crying more than three hours per day more than three days per week), demonstrated outcomes with an unvalidated diary and did not use intention to treat (ITT) analysis. None of the trials to date stand up to the scrutiny of best-practice research. The evidence is unconvincing that chiropractic care alone can provide a quick and effective treatment for infant colic.

One possible explanation as to why few interventions have been found to effectively treat colicky crying may be the failure to identify subgroups of crying babies. Research studies have by and large failed to determine whether the excessive crying stemmed from colic or from another cause [20-23]. Many chiropractors may believe that subgroups exist but have not engaged in any classification system. Such a classification system or subgrouping might be able to demonstrate improved clinical outcomes. A prospective observational study reported in 2009 [23] demonstrated improved outcomes relative to the group when 158 infants were divided into three categories, infant colic (IC) (n = 77/158 or 49%), irritable infant syndrome of musculoskeletal origin (IISMO); n = 56/158 or 35%) and inefficient feeding, crying infants with disordered sleep syndrome (IFCIDS); n = 25/158 or 16%) according to specific criteria (table 2). There were no statistically significant differences in the demographic profile of the three groups. Although the design of this study cannot determine efficacy, this was the first study of its type following babies presenting to a chiropractic clinic for excessive crying having classified the infants into three groups. Parents of excessively crying infants demonstrating a musculoskeletal problem (IISMO) reported most improvement, with colicky infants a close second, but with IIFCIDS, a syndrome of unknown origin, parents reported less improvement and more ongoing stress (Table 3) [23]. If further studies can corroborate this or another meaningful subclassification for the crying infant, better clinical outcomes may be achieved as well as improving research studies. Inclusion criteria aimed at homogenous groupings may be better able to establish efficacy.

**Table 2:** Characteristics of Colic, IISMO and IFCIDS syndromes of infancy [19,21,23]

Characteristics	Infant Colic	Irritable Infant Syndrome of Musculoskeletal origin	Inefficient feeding crying infant with disordered sleep
Common age range	2 weels-3 months; Onset may be early to late but most commonly within first 2 weeks	3 weeks to 3 months but may occur outside of these ranges, infant needs ability to hold antalgic posture	1-6 months (seen less frequently 7-12 months)
			
Crying patterns	Loud, disturbing, relentless unsoothable crying often late afternoon/evening	Crying may be high-pitched at any time of day. Often triggered by positioning child out of position of comfort	Many episodes and long bouts of crying, peaking during the day; high intensity, priercing cries common
			
Physical presentation/behaviour	Tense abdomen, flexed posture, kicking, flailing legs and boxing arms. Unconsolable whether picked up or not.	Antalgic posture held for sake of comfort; asymetric movemetns/unilateral spinal hypertonicity; tactile defensive; musculoskeletal sensitivity.	"Pained faces" (facial grimaces) accompany crying; body unrest, arching postures, general irritability and difficult to soothe; difficult to distinguish from colic crying/movements, but not limited to end of day and longer hours
			
Other signs/symptoms	Appears in pain, changes from happy to crying in an instant, wants frequent cuddling but may not respond	Restless sleep; may not wish to rest supine (some will only sleep in car seat); affective disorder common.	Male predominance (60:40); feeding problems common, sleep disorders common (difficulty falling asleep and staying asleep)

**Table 3:** Mean differences in crying, sleep and maternal stress in infant crying gorups (N = 158)[23]

Variable	Mean difference	P	95% confidence interval (CI)
Crying*			
Colic-IISMO	0.6	0.250	-0.3-1.4
Colic-IFCIDS	2.1	0.000	1.0-3.2
IISMO-IFCIDS	1.5	0.004	0.4-2.7

Sleep*			
Colic-IISMO	-4.75	0.536	-1.5-0.6
Colic-IFCIDS	1.8	0.005	0.5-3.2
IISMO-IFCIDS	2.3	0.001	0.9-3.7

Stress^##^			
Colic-IISMO	0.87	0.02	-
Colic-IFCIDS	1.3	0.06	-
IISMO-IFCIDS	0.46	0.53	-

*tukey post hoc test

^##^Man Whitney test. An analysis of variance

Until better studies can be designed and carried out, health care practitioners are faced with the dilemma as to what to recommend to parents of the excessively crying infant, a condition known to be quite dangerous to some infants as it is the leading cause of inflicted or non-accidental injury in the child [24-26]. A summary of what is known may be useful. Taking a crying baby to a chiropractor for treatment does result in fewer hours of crying but this also seems to be the case with placebo [14]. Nevertheless, there is also promise that the parent may feel less anxiety and the infant may sleep better and longer [20]. So, although controversial, we conclude that in cases where all other serious diagnoses have been excluded and in the absence of any other efficacious therapy as well as a favorable risk/benefit ratio, it seems reasonable to us to send a colicky infant for a therapeutic trial of 4-6 chiropractic treatments. Future studies require blinding the parent and the assessor and including a non-treatment control group (as in a waiting list) to determine whether chiropractic manual therapy for infant colic has more than mere promise.

### Enuresis

Enuresis, or urinary incontinence in children, is a common problem, with a prevalence that ranges from 16% at five years down to approximately 5% at 10 years of age, affecting boys twice as commonly as girls. One to two percent of children over the age of 15 will continue to have occasional nighttime urinary incontinence [27,28].

Several factors are known to play a role in nocturnal enuresis, including maturational delay, genetics, functional small bladder capacity, sleep disorders and psychological issues [29]. Other causes of nocturnal enuresis that should be considered and ruled out by the clinician include unrecognized underlying medical disorders (such as seizures, diabetes mellitus, diabetes insipidus, and hyperthyroidism), encopresis or constipation, urinary tract infection, chronic renal failure, spinal dysraphism, psychogenic polydipsia and upper airway obstruction (obstructive sleep apnea). Given the complexity and morbidity of the differential diagnosis or enuresis, the diagnostic work-up is likely beyond the scope of the average chiropractic practitioner.

A urinalysis can be helpful in evaluating for diabetes, water intoxication or occult urinary tract infection. Radiologic imaging is rarely necessary. Ultrasonography may be needed to evaluate the anatomy if there has been a history of multiple urinary tract infections or if there are significant daytime complaints as well. Neurological imaging of the spine is indicated only in children with noted abnormalities of the lower lumbosacral spine on neurological examination of the perineum or lower extremities [30].

In general, nocturnal enuresis has a very high rate of self resolution and rarely requires intervention, aside from reassurance, from a health care professional. Motivational therapy (i.e. stickers or other rewards) has been found to be successful, decreasing enuretic events by more than 80% in greater than 70% of patients [31]. Enuresis alarms are also very successful, and have been found to be more effective than the most common pharmacological therapy, tricyclic antidepressants [32]. Hypnosis has also shown some promise. Spinal manipulation seemed to give better results than sham adjustment, but the conclusions come from small trials and have not been duplicated in larger studies [33]. Although chiropractic lore has long held that enuresis responds well to chiropractic adjustments, scientific study simply does not bear this out.

### Asthma

Asthma is the most common chronic disease of childhood, affecting more than six million children in the United States, 13% of children in the United Kingdom, and 20% of children in Australia [34-36]. It is defined as an obstructive pulmonary disease characterized by reversible airway obstruction, airway inflammation with increased mucous production, and bronchial smooth muscle hyper-reactivity. That reactivity can be a response to a number of triggers, including environmental allergens such as pollens, animal danders or molds, viral upper respiratory infections, odor irritants such as cigarette smoke, occupational exposures, chemicals and dust, drugs including aspirin and non-steroidal anti-inflammatory drugs, exercise, upper airway inflammation, weather factors and gastroesophageal reflux [37-39].

Evaluating asthma can be fairly difficult. Many cases are misdiagnosed, often for years. Coughing and wheezing are the most common symptoms of childhood asthma with dyspnea, chest tightness or pressure and even chest pain commonly reported as well. Frequent cough, especially nocturnal cough, one that returns seasonally, or a cough in response to specific environmental triggers should be evaluated for a diagnosis of asthma. Many children do not present with the classic wheeze, but instead are noted to be "cough-variants" of asthma [40]. Asthma, in fact, is the most common cause of chronic cough in children older than the age of three.

Many children with asthma have an allergic history. Asthma, eczema and allergic rhinitis are often seen clustered in families and lumped into the broader category of "atopic illness." These children will frequently have elevated levels of Immunoglobulin E (IgE).

Since by definition asthma is a reversible obstructive process, the standard for diagnosis includes pulmonary function testing to prove both airway flow obstruction and reversibility. The testing is difficult in younger children, but is advised for diagnosis in children age five and older who are suspected of having asthma [41].

The mainstay of treatment for acute exacerbations of asthma is the use of inhaled beta-agonists. Short-acting beta-agonists are discouraged for frequent use between exacerbations in patients with chronic, persistent asthma, with emphasis shifting to "controller" medications, such as long-acting beta-agonists and inhaled corticosteroids. Leukotriene blockers and IgE modulators have also been shown to be successful in decreasing the frequency and severity of asthma attacks in certain subsets of patients.

Multiple studies have been done on the use of chiropractic in the treatment of asthma, with most concluding that the addition of chiropractic - while having little impact on objective markers of the disease - can lead to subjective improvement in the patient [42-45].

A recent article reporting a single case claimed improvement in asthma symptoms (a decrease in cough as reported by the patient's mother) with cessation of medication usage and demonstration of a marked increase in lung volume in a six year old girl treated with high-velocity, low-amplitude manipulations [46]. Yet another report of three cases where chiropractic manipulation administered to the upper thoracic spine twice a week for a period of six weeks was added to conventional medical therapy showed some improvement in both subjective and objective parameters [47]. These studies are in conflict with the earlier, much larger and more rigorous studies cited above, which again showed some subjective improvement, but no significant measurable change to lung function.

Many chiropractors discuss the mechanics of thoracic cage restriction and theorize that spinal manipulation improves asthma through the reduction or elimination of that restriction. While improvement in thoracic cage restriction may well improve ease of breathing, restrictive lung disorders are quite different from obstructive disorders, and therefore asthma itself will not be affected by improved thoracic mechanics. The literature does seem to indicate that while asthma itself is not impacted by the chiropractic encounter, the patient's overall quality of life and subjective symptoms are. Further research is warranted to try and help better explain and quantify this reported phenomenon.

### Otitis Media

Otitis media, or middle ear infection, is sub-divided into three separate and distinct entities. 1) Acute otitis media, which is characterized by an abrupt onset of local signs such as ear pain or pressure, and systemic signs such as malaise or fever. 2) Chronic suppurative otitis media, characterized by continuing inflammation and otorrhea, often through a perforated tympanic membrane. 3) Otitis media with effusion, often called "glue ear" which is characterized by the persistence of effusion beyond three months without signs of acute infection. Diagnosis and differentiation of the three is typically made through otoscopy with insufflation to check for appropriate movement of the tympanic membrane. Proper and rigorous training in differentiating the three is crucial, because even among pediatric residents in training in the United States, correlation between practitioners as to the accuracy of diagnosis is fairly inconsistent [48]. It is not reasonable to expect that the typical chiropractor, with very little training in otoscopy, could reliably and consistently accurately diagnose middle ear conditions.

Within the chiropractic literature, studies that differentiate between the three different forms of otitis media tend to concentrate on acute otitis media with little, if any, data having been presented on the other two forms. Therefore, this discussion concentrates on acute otitis media.

The mainstay of treatment for acute otitis media has been antibiotic therapy. While recommendations differ, with many sources including the combined American Academy of Pediatrics/American Academy of Family Practice and Centers for Disease Control and Prevention guidelines [49] recommending a "wait and see" approach, more recent guidelines are calling into question the long held belief that some 70-80% of cases of acute otitis media will self-resolve. Earlier studies upon which that number was based had less stringent enrollment criteria, and there is some thought that many of those patients had simple upper respiratory infections. The most current Agency for Healthcare Research and Quality (AHRQ) guidelines do recommend appropriate antibiotic coverage for acute otitis media, since prompt antibiotics have been shown to more rapidly resolve the signs and symptoms of acute otitis media. It has also been shown that children who receive only symptomatic treatment have higher rates of recurrence and treatment failures than children treated with antibiotics [50]. At this point, the chiropractic and manual therapy literature has little evidence beyond case reports and case series, albeit some fairly large. One randomized trial was undertaken with osteopathic full spine manipulation and it did suggest some improvement in the manipulation group. The evaluating physicians in the study were blinded, however the mothers of the patients were not, which leaves the study subject to bias [51]. At this point, there really is no credible solid evidence upon which to make recommendations regarding the use of chiropractic care in the treatment of acute otitis media.

### Attention Deficit Hyperactivity Disorder

Hippocrates gives us one of the earliest recorded descriptions of attention deficit hyperactivity disorders (ADHD) in Aphorisms. He describes it as ""quickened responses to sensory experience, but also less tenaciousness because the soul moves on quickly to the next impression." He attributed its cause to an "overbalance of fire over water" and prescribed "barley rather than wheat bread, fish rather than meat, watery drinks, and many natural and diverse physical activities."

The syndrome of ADHD is composed of three categories of symptoms: hyperactivity, impulsivity and inattention [52]. Hyperactivity may take the form of excessive fidgeting, difficulty in remaining seated when required to do so, and difficulty playing quietly. Impulsivity is typically seen in conjunction with the hyperactivity and may manifest as difficulty in waiting one's turn, being disruptive in a classroom setting, and intruding upon other's activities. Inattention may be seen as forgetfulness, easy distractibility, frequently losing or misplacing items, disorganization and poor follow through on tasks and commitments. Full diagnostic criteria are set forth in the Diagnostic and Statistical Manual [53], but a few of the core requirements include that the symptoms must be present across different settings (for example, at home and at school), they must be present prior to the age of seven and persist for greater than six months, they must impair the child's activities, be excessive for his or her developmental level, and other mental disorders must first be excluded. There are several commercially available rating systems, including the popular Connors' questionnaires which are completed by parents, teachers, and even the child if developmentally appropriate. They can be easily scored and interpreted with little prior training on the part of the clinician.

Behavioral modification and screening for learning disorders should occur early in the evaluation and treatment of ADHD, but is likely beyond the purview of most chiropractors. Psychostimulants are commonly reviled by chiropractors, yet they still remain the most effective treatment for ADHD [54]. Despite a collection of case reports and case studies, there is no good evidence of the effectiveness of chiropractic manipulation [55]. Larger, more rigorous studies are still needed before any definite recommendations can be made.

## Discussion

CO Watkins, DC, once chairman of the National Chiropractic Association, admonished chiropractors to "resolve to be bold in what we hypothesize, but cautious and humble in what we claim." The advertisements of several chiropractors, and even the literature of many of our state and national associates, make bold claims about improvements in the above conditions, among others. While there is some rather vague and contradictory data that suggests that chiropractic might have a beneficial effect on a few non-musculoskeletal conditions, to claim improvements or even "cure" is being overly optimistic to the point, at times, of outright dishonesty. More data is needed in order to make more definitive statements. Unfortunately, the majority of the new literature continues to be still more case reports and case series rather than high quality randomized controlled studies. More case reports and case series do not strengthen the case, they simply add more case reports and case series. Until this is recognized, and improved upon, our profession will continue to struggle with its credibility issues.

That is not to say that there is not a limited role for chiropractic in managing the above conditions. For the crying infant, there is some (contradictory) evidence to suggest that chiropractors may have a positive influence on this distressing problem of infancy. For enuresis, the chiropractor (if called upon) as well as the medical physician can demystify the problem and offer suggestions on behavior modification and alarms and, when appropriate, evaluate for more significant physical disorders. In asthma, as studies have shown, a positive impact on quality of life has been observed and documented in several different studies but the evidence is otherwise negative for chiropractic. Notwithstanding this, the conscientious and educated chiropractor, while working within his or her scope of practice, can potentially be a valuable member of the pediatric health care team.

## Competing interests

The authors declare that they have no competing interests.

## Authors' contributions

JM contributed the section on the crying infant. RJF contributed the other review sections. Both authors participated in drafting the discussion and both read and approved the final manuscript.
